# Image dataset: Year-long hourly façade photos of a university building

**DOI:** 10.1016/j.dib.2024.110798

**Published:** 2024-08-02

**Authors:** Marc Roca-Musach, Isabel Crespo Cabillo, Helena Coch

**Affiliations:** Architecture, Energy and Environment Research Group (AiEM) - Universitat Politècnica de Catalunya, Spain

**Keywords:** Adaptive façades, User-façade interaction, Manual control, Roller shutters, Computer vision

## Abstract

This dataset presents 9596 photos of the East and West façades of a university building in Barcelona (Spain). Images were taken every hour for one year with two identical GoPro Hero 10 cameras. The façades are composed of a grid of 28×7 windows in the East and 28×6 windows in the West. Every window has a portion of fixed glass and an operable part for natural ventilation, and mobile solar protections (roller shutters) that users can control manually. These images are of special interest due to the lack of observation data on the user-building interaction with manually controlled adaptive façades. These data are a valuable source of information for interpreting and understanding the actual use of manually controlled adaptive façades, as well as developing usage models that could be implemented in energy simulations. Also, cropping all the windows from the full-façade photos result in 1.7 M images of individual windows, which can be easily used as a computer vision and machine learning exercise to read the position of the solar protections or the operable part of the windows.

Specifications Table

**Table:** 

Subject	Architecture
Specific subject area	User interaction with manually controlled adaptive façades.
Type of data	Raw and processed images.
Data collection	Data were collected using two GoPro Hero 10 cameras, each placed in a nearby building with a direct view of the east and west façades of the case study. The camera was programmed to take photos autonomously every hour in auto-adjust mode. The camera was regularly checked by the investigators; however, due to some energy supply failures, there are some data gaps missing in the data set. A summary table is provided with the start and end timestamps of each uninterrupted period. The East façade dataset excludes night photos because it was not possible to see anything without sunlight. The dataset does not include any data from questionnaires.
Data source location	Barcelona Architecture School (ETSAB), *Universitat Politècnica de Catalunya* (Spain) (+41.38418415071659, +2.1140759035867904)
Data accessibility	Repository name: CORA.RDR Data identification number: DOI: 10.34810/data1295 Direct URL to data: https://doi.org/10.34810/data1295

## 1 Value of the Data

• These photos are a rich source of observational data as they capture the actual use of an adaptive façade that is manually controlled by the building's occupants.
• This type of data is typically challenging to acquire.
• The extensive duration of the study, spanning a full year, offers insights into the building's seasonal changes.
• The regular window grid of the façade facilitates the extraction of 1.7 M individual window images, which can be ideal for the application and development of computer vision and machine learning algorithms.
• Our dataset can be cross-referenced with the data facilitated by the university in [1], where there are real-time and historical measurement on temperature, humidity and concentration of CO2 for each classroom. This can help to analyze the correlation between indoor conditions and users’ reactions on the façade.
• This data can contribute to develop usage models of manual solar protections and windows that could be implemented on energy simulations of buildings.

## 2 Background

The motivation behind generating this dataset was to understand the user-building interaction with manually controlled solar protections [2]. Manual devices can only be controlled when people are in the building and have the will to operate them [3]. However, the reasons leading them and the energy consequences of their choices are complex to evaluate. While manual control is often studied with surveys to occupants or in-building observations [4,5], we wanted to perform an external objective observation. This approach allows us to capture the spontaneous and unfiltered behavior of the occupants, providing an accurate representation of real-world usage patterns of solar protection.

## 3 Data Description

**Fig. 1:**
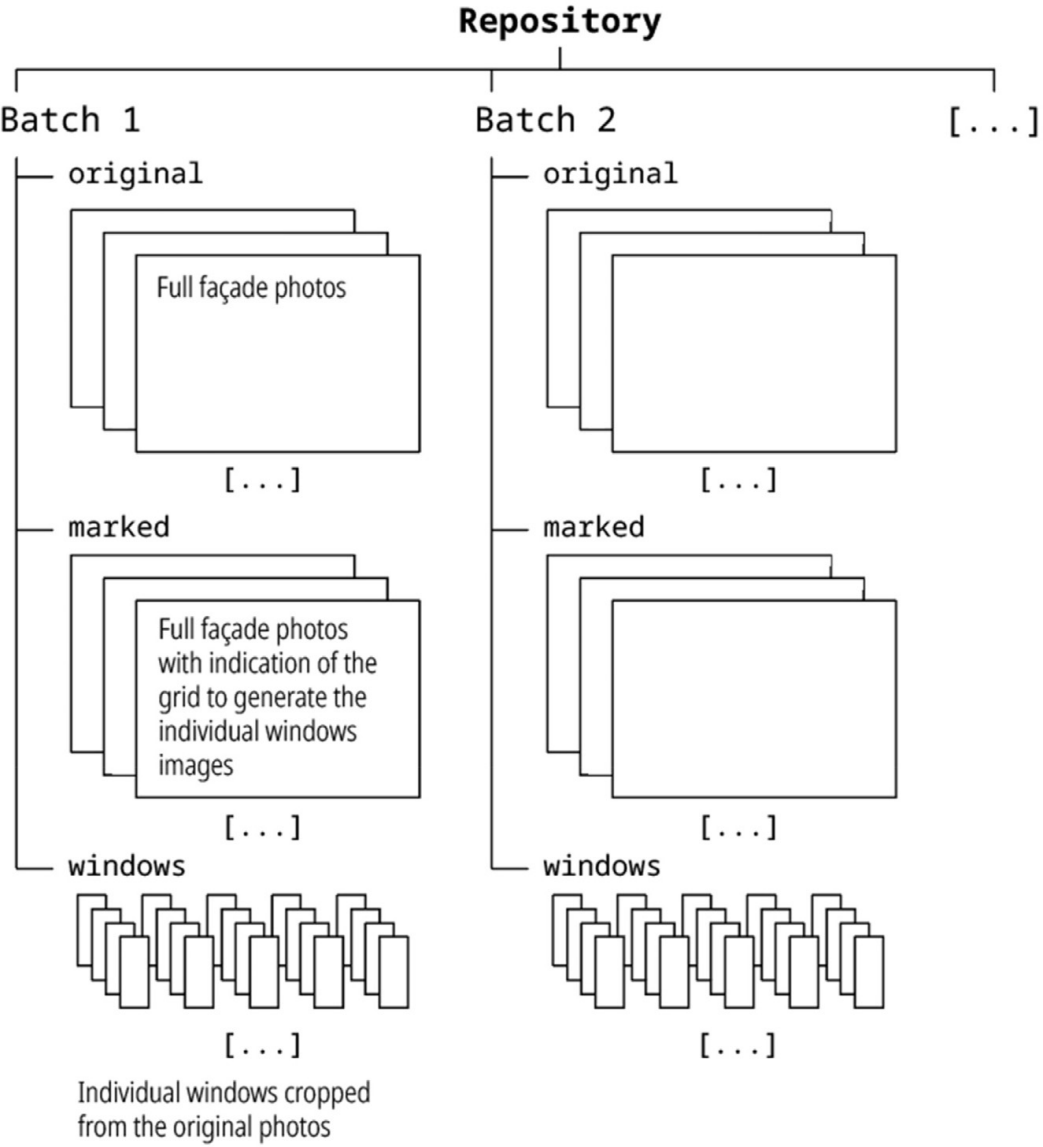
Schema of the organization of files and folders in the repository.

This dataset consists of 9596 full façade images, 6414 from the west façade and 3182 from the east, and 1.7 M processed images corresponding to the cropped individual windows from the façades. All images are in JPG format. Photos are organized (1) in batches, corresponding to uninterrupted periods when the camera was capturing photos autonomously, and (2) by type of photo. Batches are stored in separate compressed ZIP archives in the repository, and archive includes three subfolders corresponding to the different photo types: the 'original' subfolder stores the full façade photos, the 'marked' subfolder contains a file which is a graphical representation of the crop limits we used to generate windows photos from the full façade images, and the 'windows' subfolder includes the individual cropped windows. Fig. 1 shows a schema of the repository data structure of folders and files.

Batches and photo filenames follow a strict nomenclature to identify what is visible in the images and when they were taken.

**Fig. 2:**
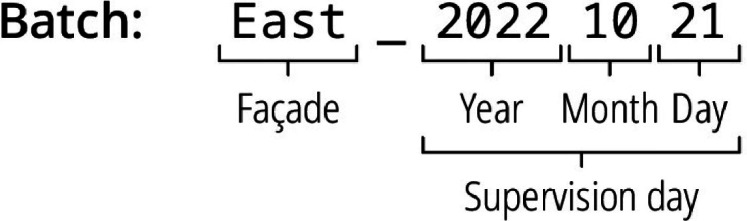
Nomenclature for batches.

The Batches nomenclature (Fig. 2) consists of the façade orientation visible on the photo (East or West), followed by the date the investigators downloaded the photos in the format YYYYMMDD. In some cases, during an uninterrupted period of photo acquisition, the camera rotated or moved for some unknown reason, which changes the alignment of the regular window grid. To facilitate postprocessing of the images, these points have been manually identified, and photos were divided into different subsets, identified by a suffix ".1", ".2" or ".3" at the end of the batch name.

**Fig. 3:**
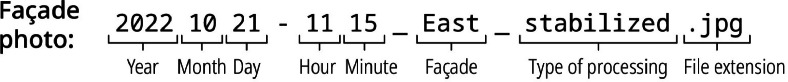
Nomenclature for the original façade photos.

**Fig. 4:**
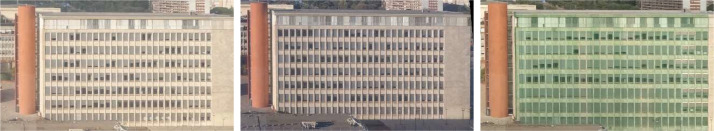
Sample of some photos of the entire façade.

Façade photos (Fig. 4) are first named with the timestamp of the photo in the format YYYYMMDD-HHMM, followed by the façade that is visible on the image (East or West), and the type of processing applied to the photo (Fig. 3). Façade photos can be *stabilized* or *marked. Stabilized* is the process used to get the general façade image from the original photo, which was cropped to suppress neighbors and surroundings for privacy reasons, the perspective was corrected to get a planar façade view, and an After Effects stabilization process was applied to ensure a more consistent position of the grid of windows. The *marked* process consists of indicating the regular grid on the *stabilized* image that we used to crop the individual window images.

**Fig. 5:**
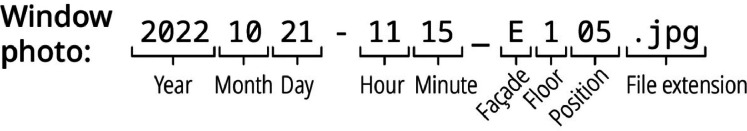
Nomenclature for window photos.

Window photos are named with the timestamp of the photo in the format YYYYMMDD-HHMM, followed by the window ID (Fig. 5).

**Fig. 6:**
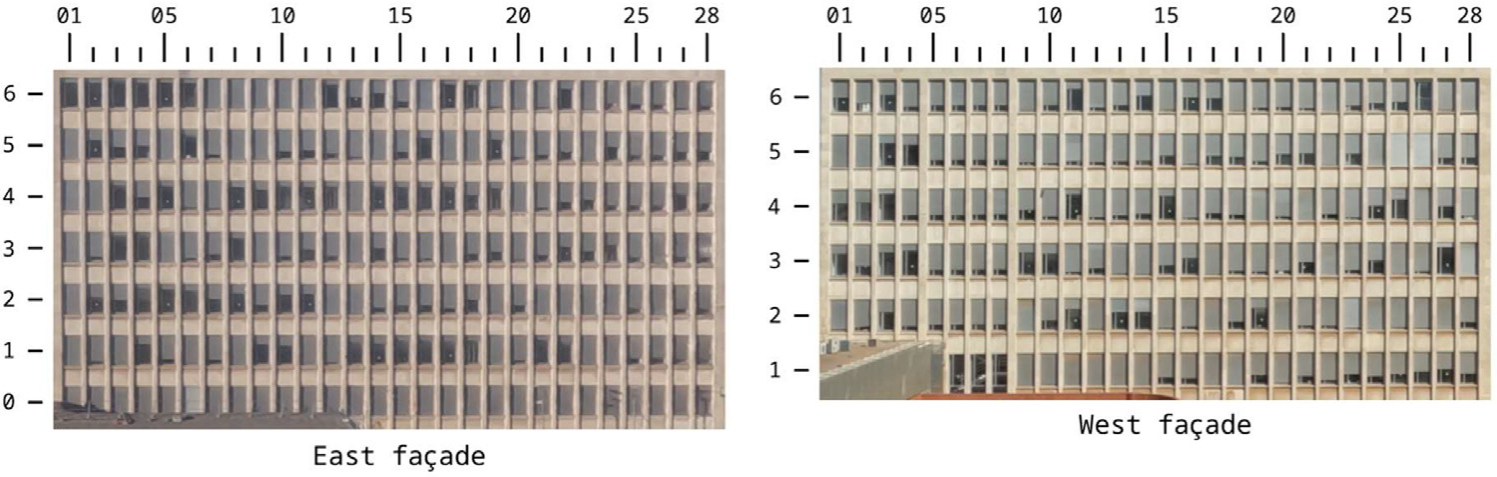
Window ID grid for the east (left) and west (right) façades.

The window ID (Fig. 6) is composed of (1) an initial letter indicating the orientation of the façade, which can be E for East or W for West; (2) a one-digit number representing the floor where the window is located, in the range 0–6, where 0 is the ground floor, 1 is the first floor, and so on; and (3) a two-digit number corresponding to the position of the window in the grid, from 01 to 28, named consecutively from left to right in the photo of the façade. The ground floor is not visible on the West façade, as shown in Fig. 6.

All façade and window images also have some EXIF metadata that provide further information about how the photo was taken. Table 1 provides a list of available metadata.

**Table 1:** Metadata for the façade and window images.

Metadata name	Example value
Camera maker	GoPro
Camera model	HERO10 Black
F-stop	f/2.5
Exposure time	1/240 s.
ISO speed	ISO-116
Focal length	3 mm
Max aperture	2.5
Metering mode	Average
Flash mode	No flash function
35 mm focal length	15
Light source	Daylight
Exposure program	Normal
Saturation	High saturation
Sharpness	Normal
White balance	Auto

Table 2 is a summary of batches, including the initial and end timestamps, the time zone of the timestamps, and the amount of façade photos and window photos per batch.

**Table 2:** Summary of batches.

Batch	Façade	First timestamp	Last timestamp	Time Zone	Façade photos	Window photos
East_20221021	East	15/10/22 16:15	21/10/22 13:15	UTC+2	70	13 720
East_20221130.1		04/11/22 14:29	19/11/22 09:29	UTC+1	146	28 616
East_20221130.2		20/11/22 08:29	30/11/22 09:29	UTC+1	105	20 580
East_20230120		30/11/22 10:15	20/01/23 11:15	UTC+1	512	100 352
East_20230531.1		17/02/23 08:15	30/03/23 09:15	UTC+1	475	93 100
East_20230531.2		30/03/23 10:15	10/05/23 14:15	UTC+1	528	103 488
East_20230531.3		10/05/23 17:15	31/05/23 14:15	UTC+1	287	56 252
East_20230712		31/05/23 16:15	12/07/23 13:15	UTC+2	670	131 320
East_20231103		30/09/23 08:15	03/11/23 17:15	UTC+2	389	76 244
West_20220905	West	29/07/22 10:15	05/09/22 10:15	UTC+2	959	156 317
West_20221021		29/09/22 11:15	21/10/22 11:15	UTC+2	529	86 227
West_20221103		21/10/22 13:15	03/11/22 11:15	UTC+2	311	50 693
West_20221130		03/11/22 14:15	30/11/22 09:15	UTC+1	644	104 972
West_20230112		30/11/22 10:15	12/01/23 10:15	UTC+1	1 028	167 564
West_20230518.1		12/01/23 11:15	16/02/23 10:15	UTC+1	706	115 078
West_20230518.2		17/02/23 20:15	27/04/23 21:15	UTC+1	1 595	259 985
West_20230614		18/05/23 17:15	14/06/23 10:15	UTC+2	642	104 646

				**TOTAL**	**9 596**	**1 669 154**

## 4 Experimental Design, Materials and Methods

**Fig. 7:**
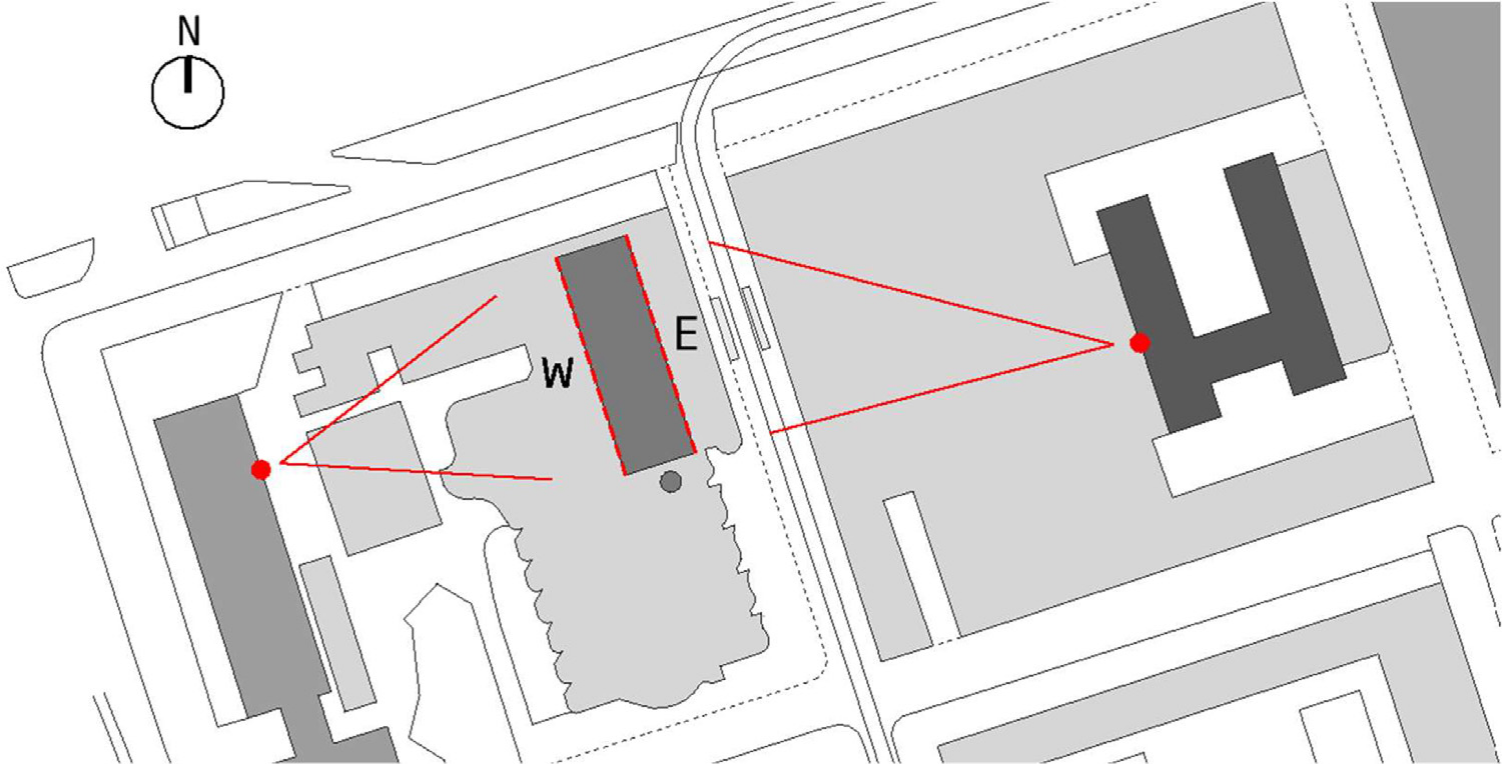
Site plan of the observed building and the position of the two cameras.

The method to take the photos was stipulated with the aim of ensuring a uniform photo acquisition and a clear view of the façades. Two equal GoPro Hero 10 cameras were placed in nearby buildings, specifically in private offices with direct view of each façade (Fig. 7).

**Fig. 8:**
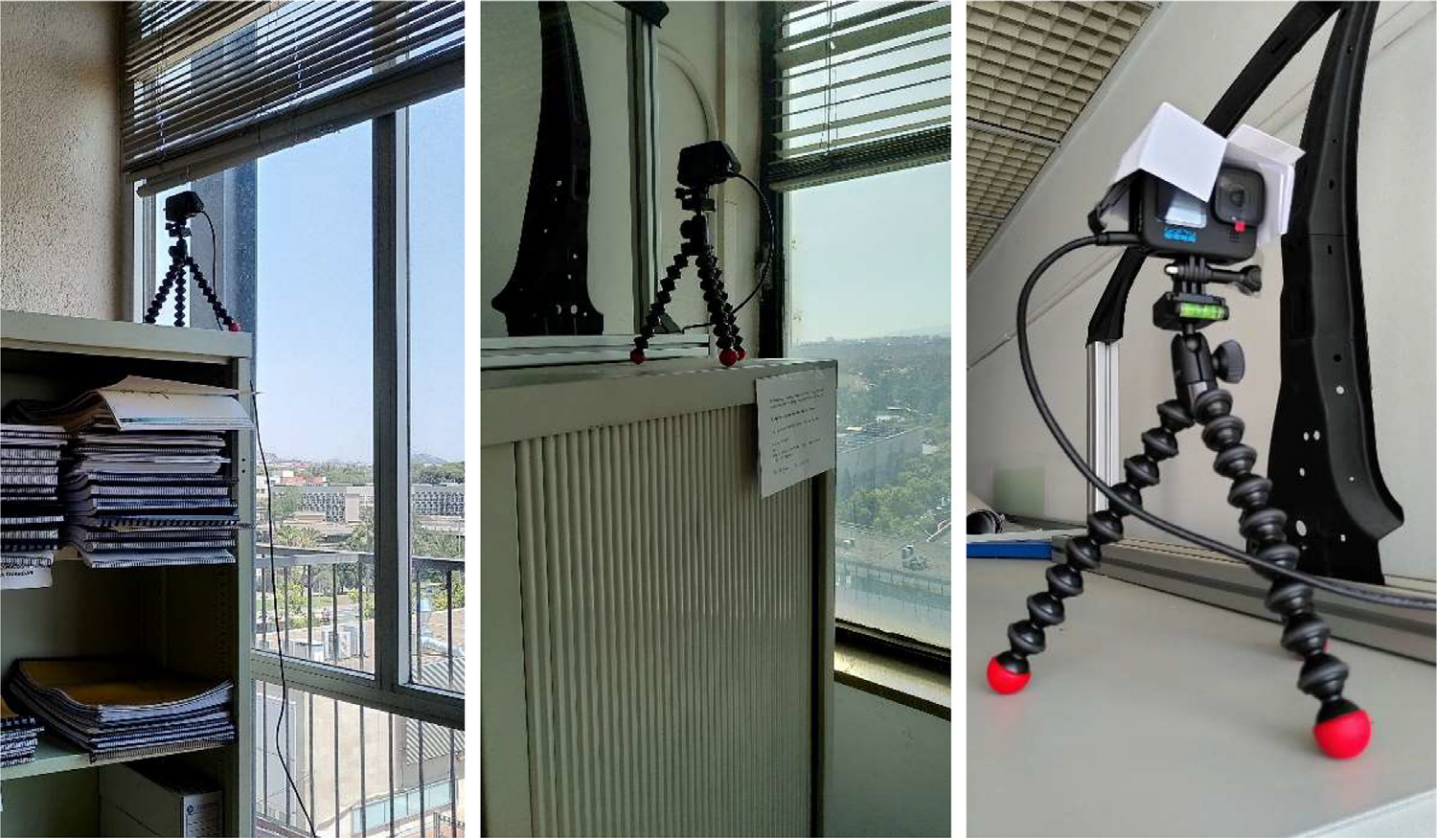
Photo of the location of the camera taking photos of the west façade (left), east façade (center), and detail of the solar protection to minimize backlit photos (right).

The cameras were placed on top of some bookshelves to minimize the possibility that someone would accidentally move the camera, as shown in Fig. 8. They were permanently plugged into the electrical grid to ensure a long period of autonomy, despite making them vulnerable to electric shortage. The office blinds were set in a position that provided solar protection for the camera, but did not interfere with the view. However, this was not enough to prevent backlit photos, so we added paper-made solar protection on top of the camera (Fig. 8).

**Fig. 9:**
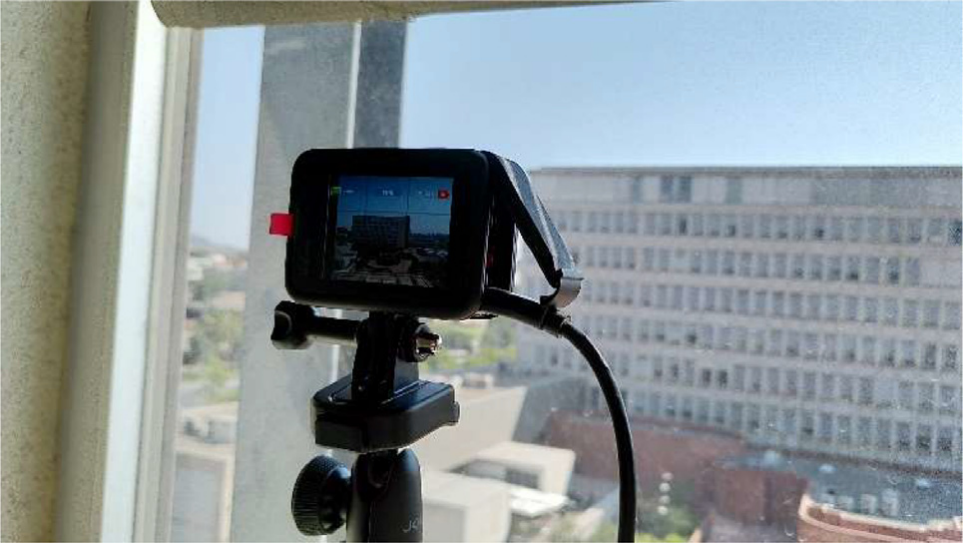
Camera view to the west façade.

Both cameras had no obstruction to see the entire façade, except for some windows on the ground floor. On the West façade it was not possible to see any window on the ground floor because there is a close-attached building that hides them (Fig. 9). On the east façade, some windows on the left of the ground floor had partial obstruction from a close building.

The camera was set in a mode to capture an unlimited sequence of photos. The only limitation was the storage capacity of the camera, so we used a 256GB SD card that had the capacity to store over one year of images. Nonetheless, the camera was supervised and images were downloaded regularly by the investigators. The frequency of supervision was approximately every month, although it was limited to access to the space where the camera was. The following checklist was established to ensure that we performed the same steps on every supervision:

1. Turn off the camera to let it cool down.
2. Extract the SD card and copy all the photos to the PC.
3. Insert the SD card and turn on the camera.
4. Adjust the date and time.
5. Check that all the façade is visible.
6. Configure again the mode to capture the sequence of photos.
7. Set the start time of the sequence of photos to the next 15 min after an o'clock hour (eg, 10:15, 11:15…)

**Fig. 10:**
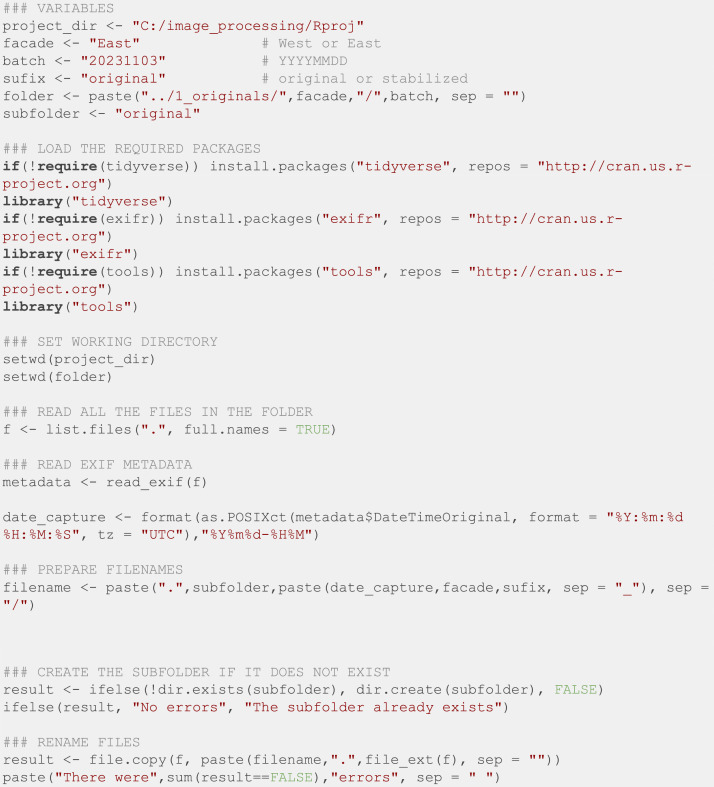
R script to set a relevant filename for every photo in a batch.

When collecting data, we stored the photos in a folder with the name of the façade followed by the date of the last image (the batch nomenclature). If, for some unexpected reason, such as an electrical shortage, the camera stopped capturing photos before the supervision day, the timestamp of the last photo was the batch date. After that, all photos were processed with an R script reading their EXIF metadata to set a human-readable filename with the relevant information for the investigation (façade and date). This script (Fig. 10) was executed on R [6] version 4.3.3, and required *tidyverse* [7] and *exifr* [8] packages.

**Fig. 11:**
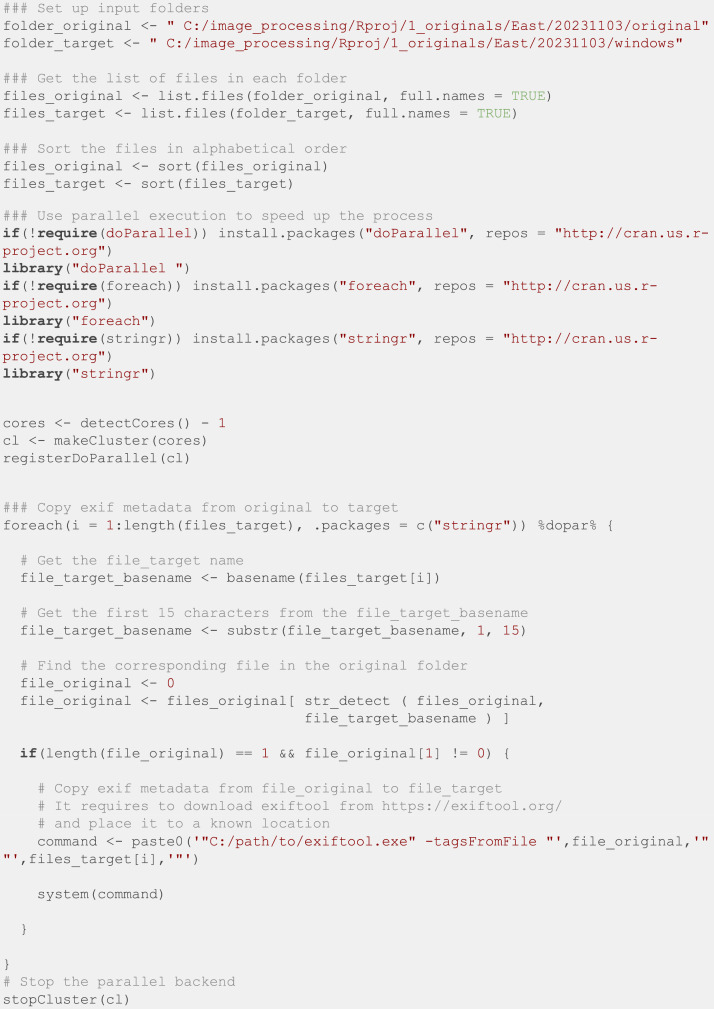
R script to restore the EXIF data using the exiftool library.

Later, all photos were processed with Adobe After Effects [9] to suppress the view from neighboring buildings, they were also aligned to a regular grid and stabilized using the Wrap Stabilizer effect combined with manual edit. Then the filenames and EXIF data were restored using the following R script (Fig. 11). This script requires the *doParallel* package [10], which takes advantage of multicore processing to speed up the script, as well as the packages *foreach* [11] and *stringr* [12].

**Fig. 12:**
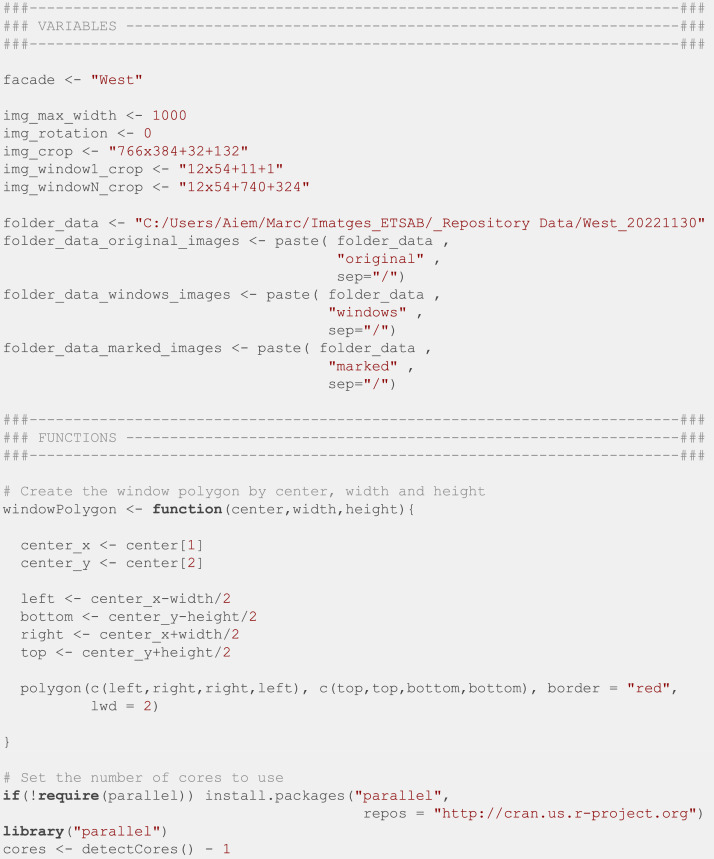

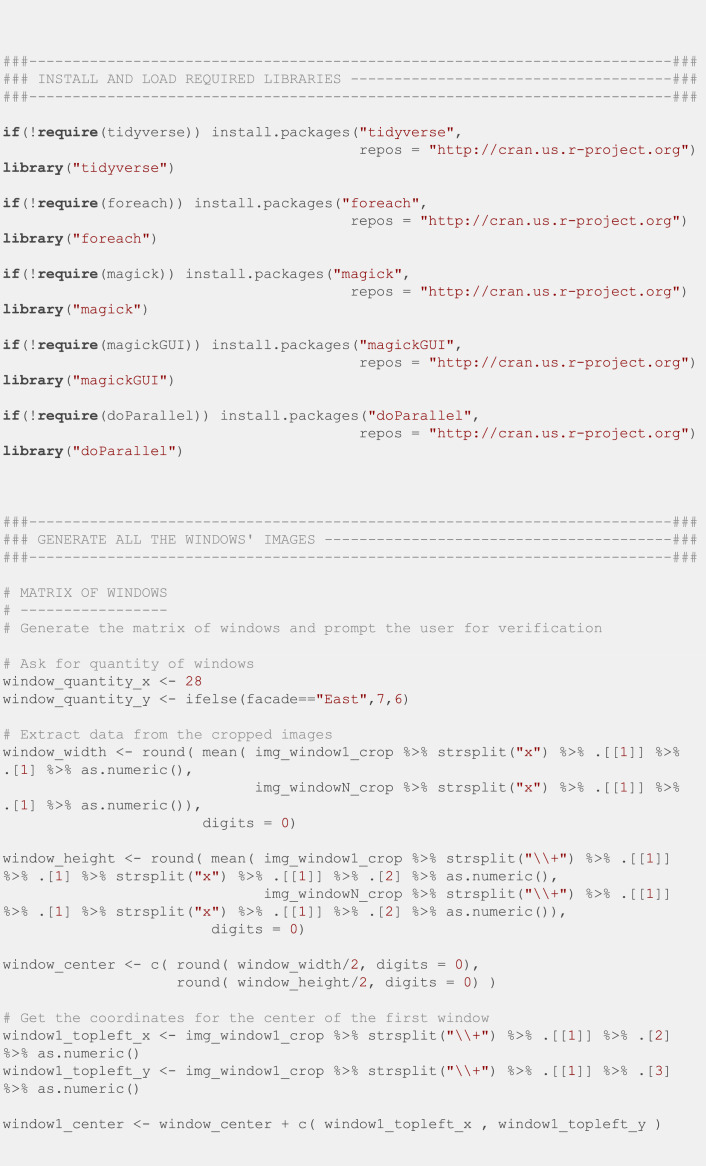

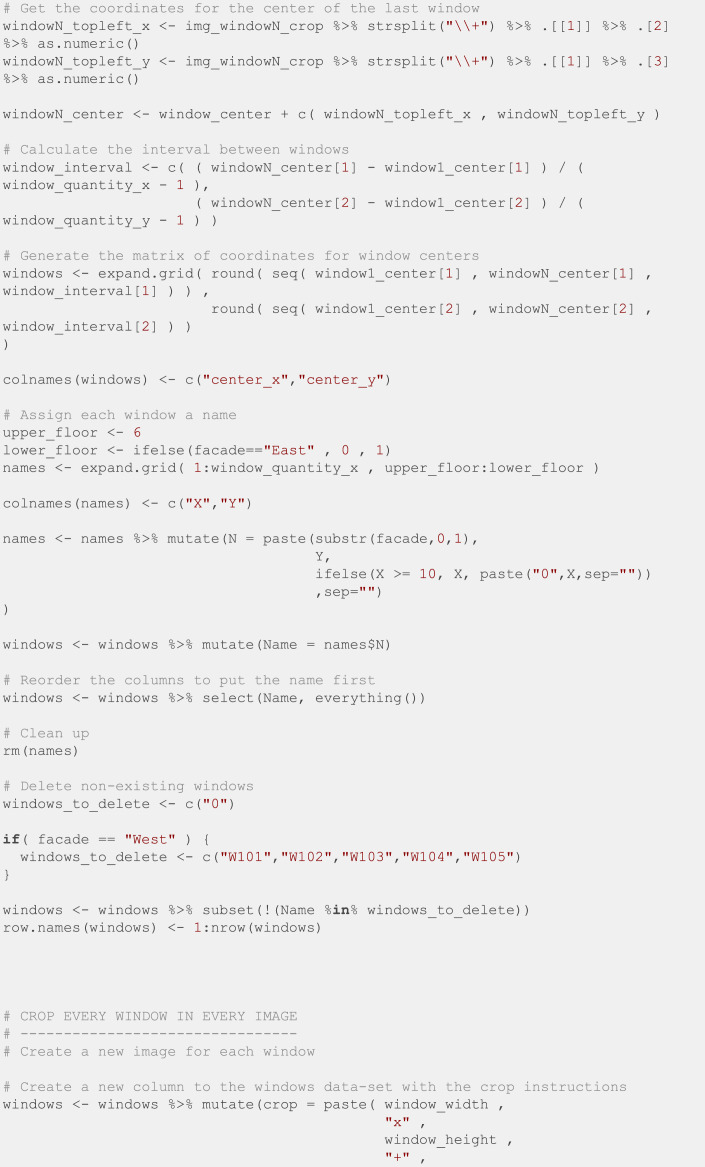

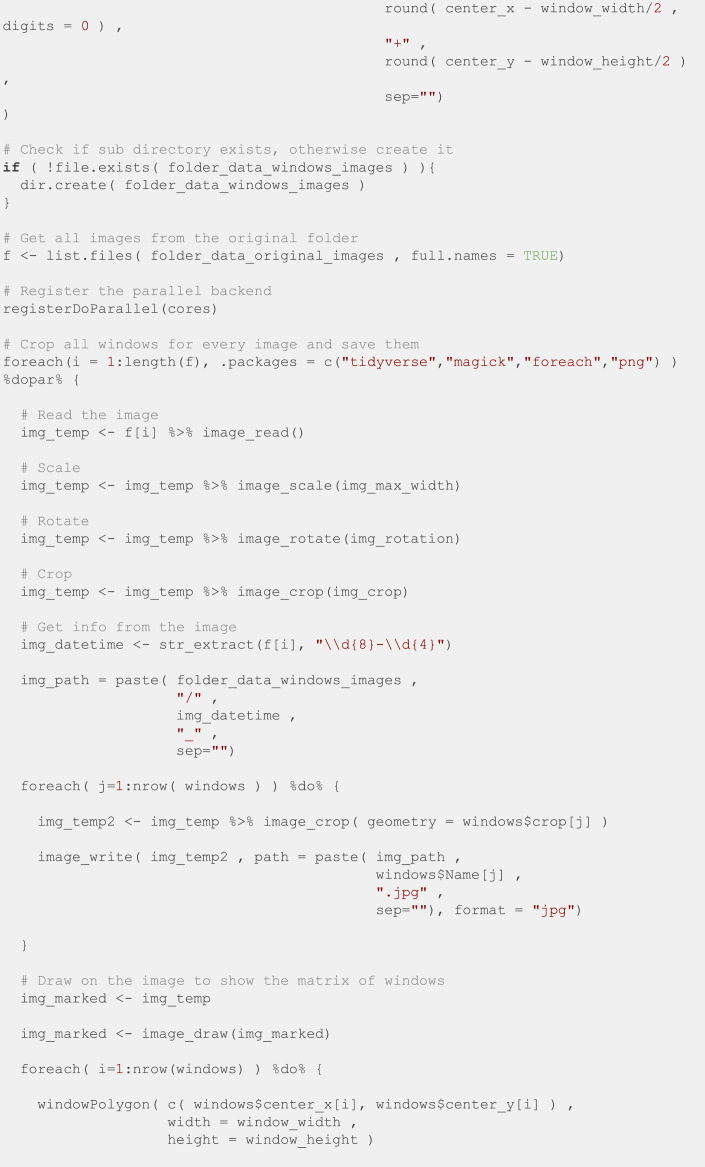

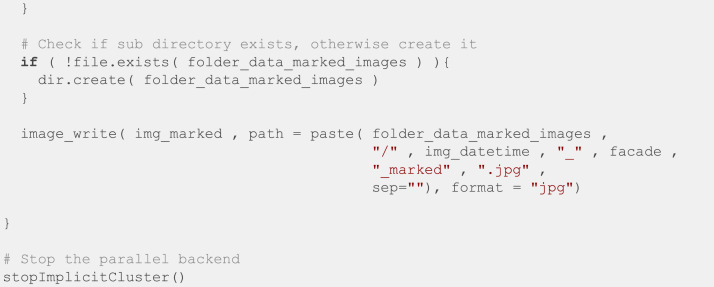
R script to crop all the windows into individual images.

Finally, we manually specified the position of the top left and bottom right windows for the first image of every batch, and we used the R script in Fig. 12 to crop all the windows into individual images. This script is based on the *magick* [13] package to manipulate images.

## Limitations

This dataset is subject to some limitations that should be considered when interpreting the data:

**Exclusion of night-time images on the East façade**: The dataset does not include night-time photos for the east. The camera captured full-black images during night periods due to the lack of illumination on the east façade. On the contrary, on the West façade there were some artificial lights on the public space that provided enough illumination to get acceptable quality images at night.

**Data interruptions:** There were a few times when the camera stopped capturing images because of electrical supply failures. The camera did not have the capability to turn on automatically when the supply was restored, so there are periods between the supervisions that the camera was not working. The data in the repository is organized by batches so that the investigators know that, within a batch, the photo series are continuous. However, between batches, there can be some gaps for hours or days, until the next supervision. If we were notified about the electrical supply failure, we went and restored the camera immediately.

**Minor camera displacements**: The supervision process required to manipulate the camera to extract the SD card and restart the capture sequence. For this reason, the camera experienced minor rotations or movements that resulted in slight variations in the framing and perspective of the photos. To correct this, we performed a post-processing alignment of the images. Also, if we detected some camera movements within a batch, we split the batch by assigning a numeric suffix (0.1, 0.2, 0.3…) at the end of the batch name.

**Daylight Saving Time (DST) adjustments**: The timestamp of the images does not always represent the local time. The camera time settings were adjusted on every supervision. However, the supervisions did not happen exactly on the same date when the DST started or ended.

**Backlit photos**: As we were capturing images all day, some of the photos were backlit during sunset or sunrise, making it difficult to recognize the façade. Furthermore, in some cases, the light reflection of the window glass puts the reflecting indoor objects in the foreground.

These limitations occur in a few images, but must be taken into consideration when analyzing the dataset.

## Ethics Statement

The authors confirm that they have read and adhered to the ethical requirements for publication in Data in Brief.

## CRediT authorship contribution statement

**Marc Roca-Musach:** Conceptualization, Methodology, Software, Investigation, Data curation, Writing – original draft, Visualization. **Isabel Crespo Cabillo:** Conceptualization, Methodology, Validation, Investigation, Writing – review & editing, Supervision. **Helena Coch:** Conceptualization, Methodology, Validation, Resources, Writing – review & editing, Supervision, Project administration, Funding acquisition.

## Data Availability

Observational dataset for one year: east and west facades of a university building in Barcelona (Original data) (CORA.RDR).
